# Acoustic communities reflects lateral hydrological connectivity in riverine floodplain similarly to macroinvertebrate communities

**DOI:** 10.1038/s41598-018-31798-4

**Published:** 2018-09-26

**Authors:** Camille Desjonquères, Fanny Rybak, Emmanuel Castella, Diego Llusia, Jérôme Sueur

**Affiliations:** 1 Institut de Systématique, Evolution, Biodiversité (ISYEB), Muséum national d’Histoire naturelle, CNRS, Sorbonne Université, EPHE, 57 rue Cuvier, 75005 Paris, France; 2 Neuro-PSI, UMR 9197, Université Paris-Sud, CNRS, Université Paris-Saclay, 91405 Orsay, France; 3 Department F.-A. Forel for Environmental and Aquatic Sciences, Earth and Environmental Science Section and Institute for Environmental Sciences, University of Geneva, Geneva, 1211 Switzerland

## Abstract

Recent studies revealed that information on ecological patterns and processes can be investigated using sounds emanating from animal communities. In freshwater environments, animal communities are strongly shaped by key ecological factors such as lateral connectivity and temperature. We predict that those ecological factors are linked to acoustic communities formed by the collection of sounds emitted underwater. To test this prediction, we deployed a passive acoustic monitoring during 15 days in six floodplain channels of the European river Rhône. The six channels differed in their temperature and level of lateral connectivity to the main river. In parallel, we assessed the macroinvertebrate communities of these six channels using classical net sampling methods. A total of 128 sound types and 142 animal taxa were inventoried revealing an important underwater diversity. This diversity, instead of being randomly distributed among the six floodplain channels, was site-specific. Generalized mixed-effects models demonstrated a strong effect of both temperature and lateral connectivity on acoustic community composition. These results, congruent with macroinvertebrate community composition, suggest that acoustic communities reflect the interactions between animal communities and their environment. Overall our study strongly supports the perspectives offered by acoustic monitoring to describe and understand ecological patterns in freshwater environments.

## Introduction

Various animals produce sound during communication, sharing information on their identity, location, physiological and behavioural condition or environment^1^. These signals are the heart of bioacoustics, a discipline that mainly aims at deciphering the modalities and functions of animal acoustic communication by understanding the emission, propagation, reception of sounds and the coding-decoding system of information^2^. At different scales of investigation, these sounds also bear information about the presence, location, abundance and species interactions. This information can be used to study the ecology of populations, communities, and landscapes. Listening to animal sounds in an ecological framework is the main perspective of ecoacoustics, a newly emerged discipline^3^. The ecoacoustics paradigm consists in using all sounds emanating from environments to monitor, describe, and study biodiversity in order to tackle fundamental and applied ecological questions such as the impact of climate change^4,5^. As such, ecoacoustics derives from bioacoustics but scales up from individuals to populations, communities, and/or landscapes to link sound and ecology.

A collection of sounds produced by a set of organisms coexisting in a given habitat over a specified time and sharing the same acoustic space constitutes an acoustic community^6^. The composition of an acoustic community relies on communication signals and sounds emitted as by-products of animal activities such as feeding, breathing, or moving. The occurrence of all these sounds in the environment is directly determined by the presence and activity of the emitters. The ecological factors conditioning the presence of species or communities in specific habitat have been investigated to a larger extend than environmental variables conditioning sound emission. A potential emitter is acoustically active only if appropriate conditions are met^4^. Not only the presence of sounds, but also sound properties, such as amplitude, repetition rate or frequency content, are also directly related to environmental variables. For example, temperature influences almost all parameters of the sounds produced by ectothermic organisms including macroinvertebrates^7^.

In freshwater habitats, the diversity and composition of macroinvertebrate communities are commonly estimated to assess the ecological quality of habitats due to the sensitivity of these organisms to stressors such as chemical pollution or temperature changes^8^. Macroinvertebrate includes the largest number of soniferous species in freshwater environments^9^. Water beetles (Coleoptera), water bugs (Hemiptera), and caddisflies (Trichoptera) are indeed known to emit sounds underwater, mostly for intraspecific communication^9^. These taxa are therefore likely to constitute a large fraction of sound sources in freshwater environments as recently testified in temperate ponds^10^. Contrary to terrestrial and marine acoustic communities that were the focus of several ecoacoustic studies^11,12^, freshwater acoustic communities have rarely been investigated.

Among freshwater habitats, European riverine floodplains are highly dynamic environments that have been largely modified by anthropic actions^13^. The main changes operated being embankments, dams and by-pass canals^14^. The river Rhône is no exception to this general European and even worldwide trend with about a third of its course (162 km out of 522 km) being artificial channels for hydro-power plants^14^. These human infrastructures have severe effects on the physical and functional properties of the river. One of the main modified environmental factors is the minimum water discharge with reductions from its natural state reaching up to several orders of magnitude^14^ (*e.g.*, 1000 m^3^.s^−1^ to 10 m^3^.s^−1^ in Lyon, France). Floodplain channels are shaped by flood disturbances^15,16^. Lateral connectivity quantifies the level of connection of the floodplain channels to the main river. Lateral connectivity varies from values close to 1, in fully connected channels flowing all year round, to values approaching 0, in fully disconnected sites. In between these two extremes, channels covering the whole spectrum of connection to the main river – from high to low flow and from connected by yearly floods to connected only by centennial floods – can be found. Variation in lateral connectivity is related to a suite of environmental factors depending upon the frequency and duration of connections with the main channel and the associated sheer stress. Such factors include, among others, flow velocity, sediment grain size, and organic content or plant development. Ultimately, lateral connectivity is found to be one of the most important determinants of macroinvertebrate community composition and turnover between floodplain sites. At low connectivity, the assemblages are dominated by lenitophilous taxa, the highest richness of Odonata and Coleoptera and the maximal representation of predators are observed. As lateral connectivity increases, so do rheophilous taxa, in particular Ephemeroptera and Trichoptera, and the representation of passive filter-feeders and plurivoltine species^15–18^.

In this study, we explored the acoustic diversity in floodplain channels and determined the links between acoustic communities and macroinvertebrate communities within the framework of varying conditions of key ecological features of these environments. We tested three predictions: (1) ecologically different freshwater environments host contrasted acoustic communities, (2) compositions of macroinvertebrate communities and of acoustic communities are strongly correlated, and (3) acoustic communities, similarly to macroinvertebrate communities, are correlated to key ecological factors, such as temperature and lateral connectivity. We tested these three predictions by coupling a passive acoustic monitoring with a classical macroinvertebrate sampling protocol in six floodplain channels of the Rhône river.

## Materials and Methods

### Study sites

Passive acoustic monitoring and macroinvertebrate sampling of freshwater communities were carried out in six secondary channels located in two reaches (Belley and Brégnier-Cordon) of the French Upper Rhône floodplain (Figure S1, Table S1). These six sites (hereafter referred to as BEAR, GRAN, MOIR, MORT, ROSS, and VILO) were chosen to account for different lateral connectivity levels (see section *Environmental variables*) among a set of 44 sites studied in the restoration program of the Rhône^14^.

### Acoustic monitoring

The sounds produced underwater in each site were monitored with an autonomous recording platform consisting of two hydrophones HTI-96 (flat frequency response between 20 Hz and 40 kHz) connected with a 20 m cable to a single digital audio field recorder SM2 (Wildlife Acoustics, 2009). The SM2 recorders were set up to record uncompressed .wav audio files at a 44.1 kHz sampling frequency and a 16 bit digitization depth. To capture most of the sites acoustic composition, we used two hydrophones placed 6.3 +/− 2.1 m away from each other and attached underwater to a stake at 0.18 +/− 0.07 m above the sediment, with their piezoelectric element directed downward toward the sediment. The recording schedule was set to 1 min per hour, 24 hours a day. The acoustic monitoring lasted 15 days, from the 20^th^ of June 2014 to the 4^th^ of July 2014, resulting in 4,320 one-minute audio files. To avoid weather disturbances, such as rain or wind, that could impair acoustic analyses, five days of recordings with similar stable weather conditions were selected across the study period (*i.e*., 20/06/2014, 22/06/2014, 26/06/2014, 01/07/2014 and 04/07/2014) for further analyses. These five days, that resulted in a subset of 1,440 one-minute files (6 sites × 2 hydrophones × 24 hours × 5 days), were selected based on wind speed and rainfall measurements collected at two weather stations from the Réseau d’Observation Météo du Massif Alpin (ROMMA, http://www.romma.fr/) located in Brégnier-Cordon (45°38′05′′N, 05°37′13′′E) and Chrindrieux (45°49′18′′N, 05°51′05′′E).

### Assessment of the composition of the acoustic communities

The subset of 1,440 one-minute files was analysed in a random order by aural listening and visual inspection of oscillograms and spectrograms with the audio software Audacity (version 2.0.5; spectrogram parameters: Fourier window length: 512 samples; frame overlap: 0%; and window type: Hanning). This analysis focused on the detection of sound events, *i.e*., any substantial shift in sound amplitude over background noise showing a singular acoustic structure, expected to be produced by freshwater species or other biotic sources such as gas exchanges due to plant respiration^19^. Since no sound reference exists for most freshwater species (except anurans), a direct link between a particular sound event and a species cannot be made and thus species identification was not conducted. Each sound event in each recording was time delimited and allocated to a sound type according to its temporal and spectral properties (*e.g*., sound duration, dominant frequency, frequency modulation). The allocation of sound types was re-evaluated at the end of the annotation process to make sure sound types were well defined. Similar sound types, that is with overlapping frequency, duration and temporal structure, were merged. This re-evaluation reduced the number of sound types from 139 to 128.

Moreover, each sound type was assigned to one of the seven following categories: (1) *pure tone*: continuous sound lasting more than 0.1 s with a frequency band narrower than 500 Hz; (2) *noisy sound*: continuous sound lasting more than 0.1 s with a frequency band broader than 500 Hz; (3) *simple pulse*: sound lasting less than 0.1 s; (4) *composed pulse*: sound composed of several simple pulses; (5) *harmonic sound*: continuous sound with harmonics; (6) *irregular sound*: sound without a clear pattern; and (7) *composed sound*: complex sound composed of at least two of the previous categories, typically two of the previous categories. All the sound types were described by measuring the acoustic properties of a random subset of sound events (n = 1–6 sound event per sound type): the dominant frequency for tonal sound types and instantaneous frequency for non-tonal sound types and duration were measured using Audacity with a 12 Hz and 1 ms precision respectively.

The sound annotation process resulted in a presence-absence matrix of sound types across the recordings determining the sound type composition of each recording. This presence-absence matrix was subsequently used for multivariate analysis of the acoustic community composition.

### Macroinvertebrate sampling

The macroinvertebrate sampling consisted in six benthic samples per site: three collected during spring (between the 17^th^ of March and the 3^rd^ of April 2014) and three during summer (between the 7^th^ and 10^th^ of July 2014); leading to a total of 36 samples. These periods ensured to collect most of species as larvae or adults and to avoid the recording period. Individual samples were collected during the day (between 9 am and 6 pm) at random locations within the sites by thoroughly sweeping a hand net (mesh size: 500 µm²) within a 0.5 × 0.5 m metal frame. The material collected was preserved in ethanol and sorted in the laboratory. Macroinvertebrates were subsequently identified to the finest possible taxonomic level, usually species or genus.

### Composition of acoustic communities and of macroinvertebrate communities

To characterise acoustic and macroinvertebrate communities, we used multivariate analyses revealing differences in composition between the six sites.

The hourly presence-absence matrix of sound types, composed of 1,440 rows (number of analysed files) and 128 columns (number of sound types) was reduced to a daily presence-absence matrix per site of 30 rows (5 days × 6 sites) and 128 columns (number of sound types) to compare the composition of the acoustic communities among the six studied sites. To reduce the matrix, the information provided by the two hydrophones within each site was pooled together. This database was then grouped daily, transforming the hourly presence-absence matrix into a daily presence-absence matrix of sound types composed of 30 rows (5 days × 6 sites). Then, this daily matrix was treated with a Correspondence Analysis (CA), which is the appropriate multivariate analysis for presence/absence data^20^. The results of this CA were processed with a between-class Correspondence Analysis (bCA) using sites as a factor of variance maximization.

An abundance matrix of macroinvertebrate taxa, composed of 36 rows (number of macroinvertebrate samples) and 142 columns (number of macroinvertebrate taxa identified) was built to compare the composition of the macroinvertebrate communities among the six sites. Then, this abundance matrix was treated with a Principal Correspondence Analysis (PCA), which is the appropriate multivariate analysis for abundance data^18^. The results of this PCA were processed with a between-class Principal Correspondence Analysis (bPCA) using sites as a factor of variance maximization.

The first three axes of these between-class analyses were used to: (1) visualize the differences in community composition between sites; (2) identify the sound types or macroinvertebrate taxa driving these differences; and (3) study the relationship between the community composition and the environmental variables. Thanks to the between-class analysis, sites or samples with similar sound type or macroinvertebrate compositions appear close in the multivariate space.

### Environmental variables

To test whether the composition of the acoustic communities and the composition of the macroinvertebrate communities were related to the main environmental variables, water temperature and lateral connectivity were estimated at each site (Table S1).

A water temperature sensor (Tidbit® v2, Onset, Bourne, MA, USA) was attached to a submerged stake next to each hydrophone. The 12 sensors recorded water temperature every hour in phase with the acoustic recordings. The hourly temperature was extracted for the five selected days. Two variables for temperature were computed to disentangle the intra and inter-site variation of temperature. The site temperature was calculated as the average temperature over the study period for each site in order to assess the inter-site variation of temperature. The daily deviation of temperature was then calculated to assess the intra-site variation of temperature by subtracting the average site temperature per day from the average site temperature.

Indirect measures of lateral connectivity were introduced in previous studies to reduce the cost of monitoring year-round the connection of each site to the main river and the drag forces applied to the sites^21^. Specifically, lateral connectivity was estimated with the index described in Paillex *et al*. (2007). This connectivity index was shown to be a suitable proxy of connection frequency and flood disturbance regime in the study channels^22^. The calculation of the index is based on four environmental variables: (i) the organic matter content of the top 5 cm of the sediment, measured by weight loss on ignition; (ii) the electrical conductivity (µSiemens.cm^−1^) of the water; (iii) the dimensionless Simpson diversity of the mineral sediment composition calculated over four categories (clay + silt/sand/gravels/pebbles); and (iv) the horizontal cover by submerged vegetation. The four variables measured for all the 44 sites of the restoration program and for all the sampling years were processed in a standardized PCA. The index of connectivity was made up with the scores of the sites on the first axis of the PCA scaled between 0 and 1^21^ (lowest and highest connectivity respectively).

### Link between acoustic and macroinvertebrate composition and environmental variables

Two sets of three Generalized Linear mixed models (GLMM) with Gaussian structure and identity link function were used to assess the link between acoustic composition and environmental variables (average site temperature, daily deviation in temperature and lateral connectivity), and the link between macroinvertebrate composition and environmental variables (average site temperature and lateral connectivity). The response variables for the GLMM models were either the three first bCA axes characterizing the acoustic composition, or the three first bPCA axes characterizing the macroinvertebrate composition. Average site temperature and lateral connectivity were included as fixed effects, and site and date as random effects. Daily deviation in temperature per site was included as a fixed effect only in the models for acoustic community composition as it was not meaningful for macroinvertebrate community composition. To keep type I error at the nominal level of 5%, all required random slopes were also included^23^. Site temperature, daily temperature, and lateral connectivity were approximately symmetrically distributed. The environmental variables were z-transformed (mean of zero and a standard deviation of one) to reduce the chance of obtaining a non-converging model. The model was fitted in R^24^ using the function *lmer* of the R-package *lme4*^25^ (version 1.1.10). The assumptions of normality and homogeneity of the residuals were checked by visually inspecting a quantile-quantile plot and the residuals against the fitted values, both indicating no deviation from these assumptions. Model stability was checked by excluding data points one at a time from the data. Variance inflation factors^26^ were derived using the function *vif* of the R-package *car*^27^ (version 2.1.0) applied to a standard linear model excluding the random effects and did not indicate collinearity between fixed effects to be an issue. The full model was compared with the null model (*e.g*., excluding all the predictors or the predictor tested) to test the model and predictors significance.

## Results

### Characteristics of freshwater acoustic communities

**Figure 1:**
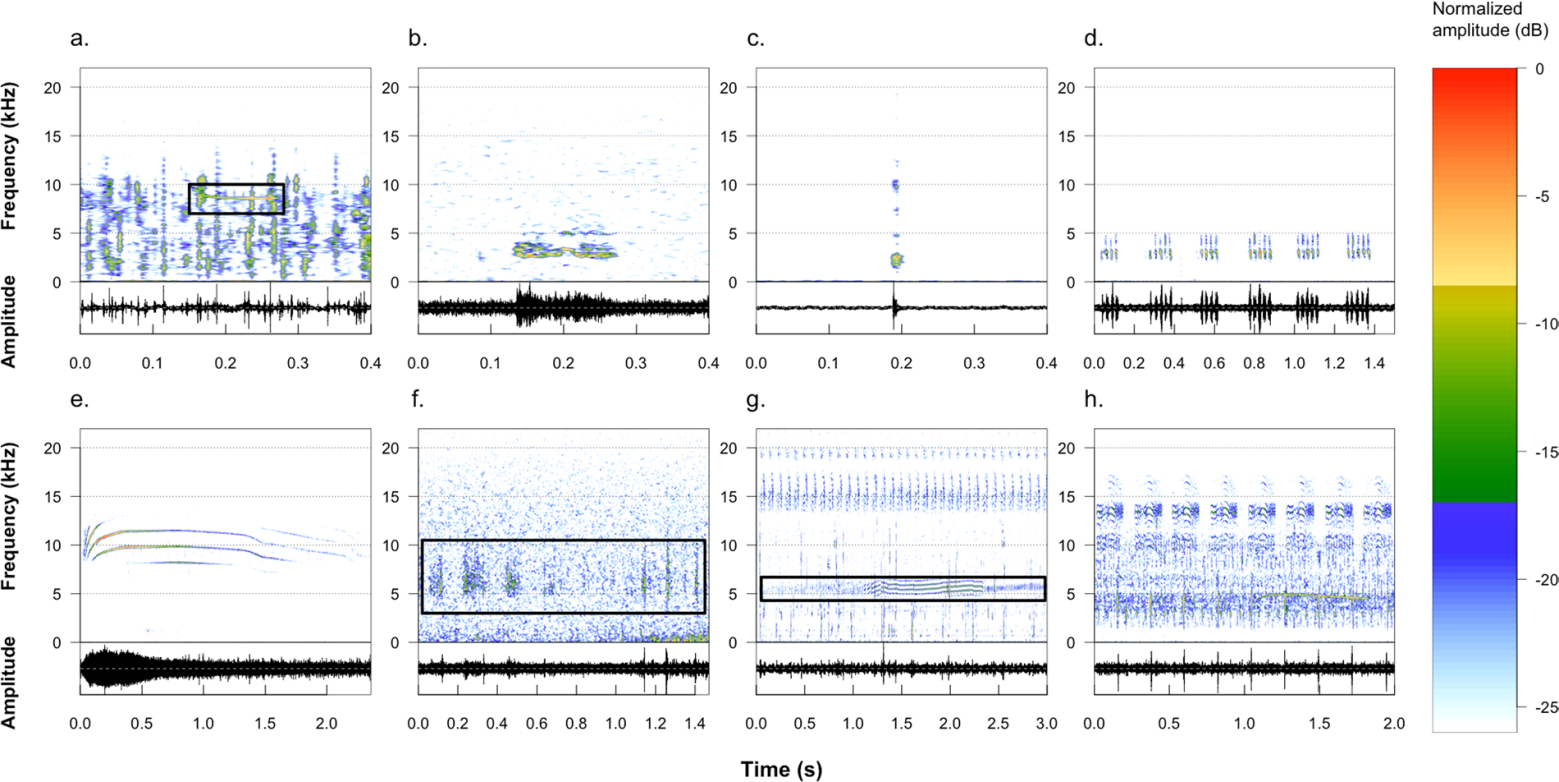
Spectrograms and oscillograms of an example of each of the seven sound categories and of one recording containing several categories (Fourier window length: 512 samples, frame overlap: 50%, window type: Hanning): (**a**) sound type 104, *pure tone*; (**b**) sound type 103, *noisy sound*; (**c**) sound type 75, *simple pulse*; (**d**) sound type 1, *composed pulses*; (**e**) sound type 50, *harmonic sound*; (**f**) sound type 63, *irregular sound*; (**g**) sound type 118, *composed sound*; and (**h**) recording from MORT on the 26^th^ of June at 12:00 am.

A total of 128 sound types were identified (Table S2). The sound types had a mean duration of 1.14 +/− 2.37 s and an average dominant frequency of 5462 +/− 4247 Hz (Table 1). Half of the sound types had their dominant frequency between 2300 and 8800 Hz (Table 1). The seven categories of sound types characterized by different duration and frequency characteristics (Fig. 1, Figure S2) exhibited different diversity and abundance (Table 1). The category of *composed pulses* was the most diverse (45 sound types) across the studied acoustic communities, whereas the *simple pulses* category was the least diverse (7 sound types). *Irregular sounds* and *simple pulses* were the most commonly recorded categories, whereas *composed sounds* and *pure tones* were the least abundant. *Simple pulses* had the shortest average duration (0.027 +/− 0.061 s) and *irregular sounds* the longest (2.978 +/− 4.091 s). *Irregular sounds* had the lowest average dominant frequency (2210 +/− 3233 Hz) and *harmonic sounds* the highest (7314 +/− 4124 Hz).

**Table 1:** Diversity, abundance and acoustic characteristics of the seven categories of sound types: number of sound types in each category; average number of times a sound type occurs in each category given as mean +/− s.d.; dominant frequency (Hz) and duration (s) given as mean +/− s.d.

Category	Number of sound types	Average abundance	Dominant frequency (Hz)	Duration (s)
1. Pure tone	12	5.7 (+/−7.4)	5608 (+/−4158)	0.74 (+/−1.56)
2. Noisy sound	24	20.2 (+/−41.3)	3989 (+/−4493)	0.83 (+/−2.15)
3. Simple pulse	7	312.9 (+/−378.9)	5457 (+/−3985)	0.03 (+/−0.06)
4. Composed pulse	45	13.4 (+/−33.2)	6549 (+/−3889)	0.84 (+/−1.91)
5. Harmonic sound	18	19.4 (+/−37.0)	7314 (+/−4125)	0.50 (+/−0.76)
6. Irregular sound	12	373.4 (+/−411.7)	2210 (+/−3233)	2.98 (+/−4.09)
7. Composed sound	10	5.0 (+/−7.5)	5526 (+/−3353)	2.84 (+/−2.63)
Total	128	64.3 (+/−192.2)	5462 (+/−4247)	1.14 (+/−2.37)

Categories refer to: (1) *pure tone*: continuous sound lasting more than 0.1 s with a frequency band narrower than 500 Hz; (2) *noisy sound:* continuous sound lasting more than 0.1 s with a frequency band broader than 500 Hz; (3) *simple pulse*: sound lasting less than 0.1 s; (4) *composed pulse*: sound composed of several simple pulses; (5) *harmonic sound*: continuous sound with harmonics; (6) *irregular sound*: sound without a clear pattern throughout; and (7) *composed sound*: complex sound composed of at least two of the previous categories.

### Characteristics of freshwater macroinvertebrate communities

142 macroinvertebrate taxa were identified to the species (78), genus (40) or family (24) level (Table S3). Coleoptera were the most diverse taxa with 40 taxa accounting for 27% of the total richness and were present in all the sites. The least diverse higher taxa were Hydrachnidia, Megaloptera, Plecoptera and Isopoda with one taxa each. Plecoptera was present only in MOIR. BEAR was the site with the highest number (fifteen) of taxa only found in this site.

### Composition of acoustic communities across the six floodplain channels

Acoustic communities were characterized by a high variability in sound types showing a site-specific acoustic composition. Only 19 sound types (15%) were found in all the studied sites. An average of 29 +/− 8 different sound types where found per day in each site.

**Figure 2:**
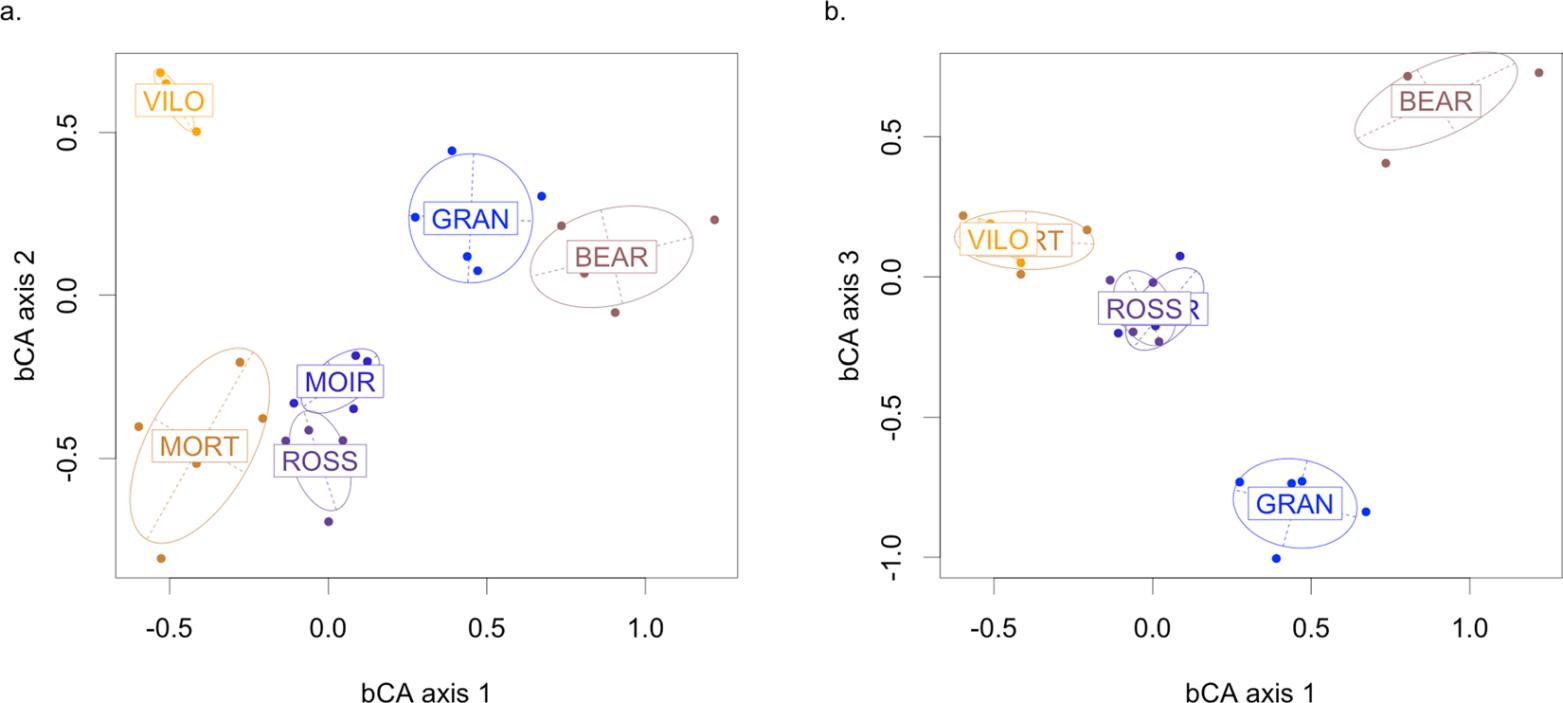
Between-class Correspondence Analysis (bCA) applied to the composition of the acoustic communities. The sites were used as factors for variance maximization. The plots (**a**) and (**b**) are projections of the composition of the acoustic communities on the first three axes of the bCA. Each point corresponds to the composition of the acoustic community recorded at one site during one day. The distance between points indicates acoustic composition dissimilarity. The dispersion ellipses surround the position of an acoustic community providing an index of the dispersion around the centroid (67% of the acoustic compositions are expected to be in the associated ellipse).

The bCA of the composition matrix revealed a significant difference in sound type composition between the sites (permutation test: 1000 permutations, p-value < 0.001, Fig. 2). The first three axes explained 73.3% of the overall variance (first axis: 29.4%, second axis: 22.9%, third axis: 21.0%). The coordinates of the sites in the three first bCA axes revealed BEAR as the most distant site from the other sites (Fig. 2). The ordination of the sites was best explained by the positive contributions of one *composed sound* (48), one *composed pulse* (56), and two *pure tones* (65 and 67) to the first axis, the positive contribution of three *noisy sounds* (4, 99, and 107) and three *composed sounds* (112, 118 and 128) to the second axis, and the negative contribution of a diverse group of sounds (76, 81, 83, 93, 101, 115, and 117) to the third axis. Among these influential sound types, none were in the categories 3 (*simple pulses*) or 6 (*irregular sounds*), which had the highest average abundance and were mostly common to all the sites (Table 1). These results suggest that the most common categories of sounds are the least suited for soundscape description, here *simple pulses* and *irregular sounds*.

### Composition of macroinvertebrate communities across the six floodplain channels

The macroinvertebrate communities were also characterized by a high variability in macroinvertebrate taxa, showing a site-specific macroinvertebrate composition. Only 18 taxa (13%) were found in all the studied sites. An average of 29 +/− 7 different taxa where found per sample in each site.

**Figure 3:**
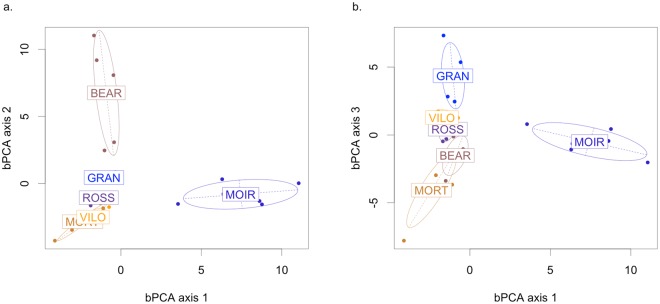
Between-class Principal Component Analysis (bPCA) applied to the composition of the macroinvertebrate communities. The sites were used as factors for variance maximization. The plots (**a**) and (**b**) are projections of the composition of the macroinvertebrate communities on the first three axes of the bPCA. Each point corresponds to a sample of macroinvertebrate in one site. The distance between points indicates macroinvertebrate composition dissimilarity. The dispersion ellipses surround the position of a macroinvertebrate community providing an index of the dispersion around the centroid (67% of the macroinvertebrate compositions are expected to be in the associated ellipse).

For macroinvertebrates, the bPCA of the abundance matrix also revealed a significant difference in taxonomic composition between the sites (permutation test: 1000 permutations, p-value < 0.001, Fig. 3). The first three axes explained 77.3% of the overall variance (first axis: 30.9%, second axis: 28.2%, third axis: 18.2%). The ordination of the sites was best explained by the positive contributions of two Gastropods (*Potamopyrgus antipodarum* and *Haitia acuta*) to the first axis, the positive contribution of a Gastropod (*Anisus vortex*) and an Odonata (*Pyrrhosoma nymphula*) to the second axis, and the negative contribution of an Hirudinea (*Alboglossiphonia* sp.) and the positive contribution of a Trichoptera (*Athripsodes aterrimus*) to the third axis.

### Environmental characteristics of the sites

The water temperature differed significantly between the six sites, with MOIR being the coldest site (12.5 +/− 0.7 °C) and MORT the warmest site (19.7 +/− 1.2 °C; ANOVA on mean daily temperatures: F_(5,24)_ = 83.13, p-value < 0.001, Table S1). The daily deviations from the average temperature ranged from −1.61 °C to 0.89 °C.

The first axis of the PCA, used to assess connectivity, explained 62.9% of the total variability. The order of increasing lateral connectivity of the sites was VILO, MORT, BEAR, ROSS, MOIR, GRAN (Table S1).

### Link between acoustic composition and environmental variables

The sound type composition explained by the first and second bCA axes was not linked to any of the environmental variables, as shown by the GLMMs testing the acoustic composition in relation with average site temperature, and daily temperature deviation, and lateral connectivity (overall model significance for the first bCA axis: df = 3, χ^2^ = 2.19, p-value = 0.53; and for the second bCA axis: df = 3, χ^2^ = 0.27, p-value = 0.96; Table 2). In contrast, the third bCA axis was significantly correlated with lateral connectivity (df = 1, χ^2^ = 10.20, p-value < 0.01, Table 2).

**Table 2:** Results of the six GLMMs based on acoustic communities.

Response variable	Term	Estimate	Standard error	Statistics (χ^2^)	Degrees of freedom	P-value
Axis 1	Intercept	0.07	0.16	^(1)^	^(1)^	^(1)^
	Lateral connectivity	0.06	0.22	0.08	1	0.776
	Average temperature	−0.22	0.22	0.86	1	0.344
	Daily temperature deviation	0.008	0.02	0.11	1	0.740
Axis 1 excluding BEAR	Intercept	−0.35	0.26	^(1)^	^(1)^	^(1)^
	Lateral connectivity	0.34	0.04	14.74	1	0.0001***
	Average temperature	0.02	0.01	0.77	1	0.38
	Daily temperature deviation	0.03	0.03	0.68	1	0.41
Axis 2	Intercept	−0.28	1.50	^(1)^	^(1)^	^(1)^
	Lateral connectivity	0.07	0.22	0.09	1	0.76
	Average temperature	0.02	0.09	0.05	1	0.83
	Daily temperature deviation	−0.02	0.04	0.16	1	0.69
Axis 2 excluding BEAR	Intercept	−0.08	1.82	^(1)^	^(1)^	^(1)^
	Lateral connectivity	0.03	0.26	0.02	1	0.90
	Average temperature	0.009	0.11	0.008	1	0.93
	Daily temperature deviation	−0.03	0.04	0.45	1	0.50
Axis 3	Intercept	0.02	0.07	^(1)^	^(1)^	^(1)^
	Lateral connectivity	−0.50	0.09	10.20	1	0.001**
	Average temperature	−0.19	0.09	2.12	1	0.15
	Daily temperature deviation	−0.05	0.05	0.96	1	0.33
Axis 3 excluding BEAR	Intercept	−0.10	0.04	^(1)^	^(1)^	^(1)^
	Lateral connectivity	−0.40	0.05	12.33	1	0.0004***
	Average temperature	−0.13	0.05	3.94	1	0.047*
	Daily temperature deviation	−0.02	0.02	0.29	1	0.59

For each model and each term in the models, the estimate, the standard error, the χ^2^, the number of degrees of freedom and the p-values are reported, except for intercepts (p-value * < 0.05, ** < 0.01, *** < 0.001). For statistical details, see subsection *Link between acoustic and macroinvertebrate composition and environmental variables* of the Materials and Methods.

^(1)^Not shown due to the lack of meaningful interpretation.

**Figure 4:**
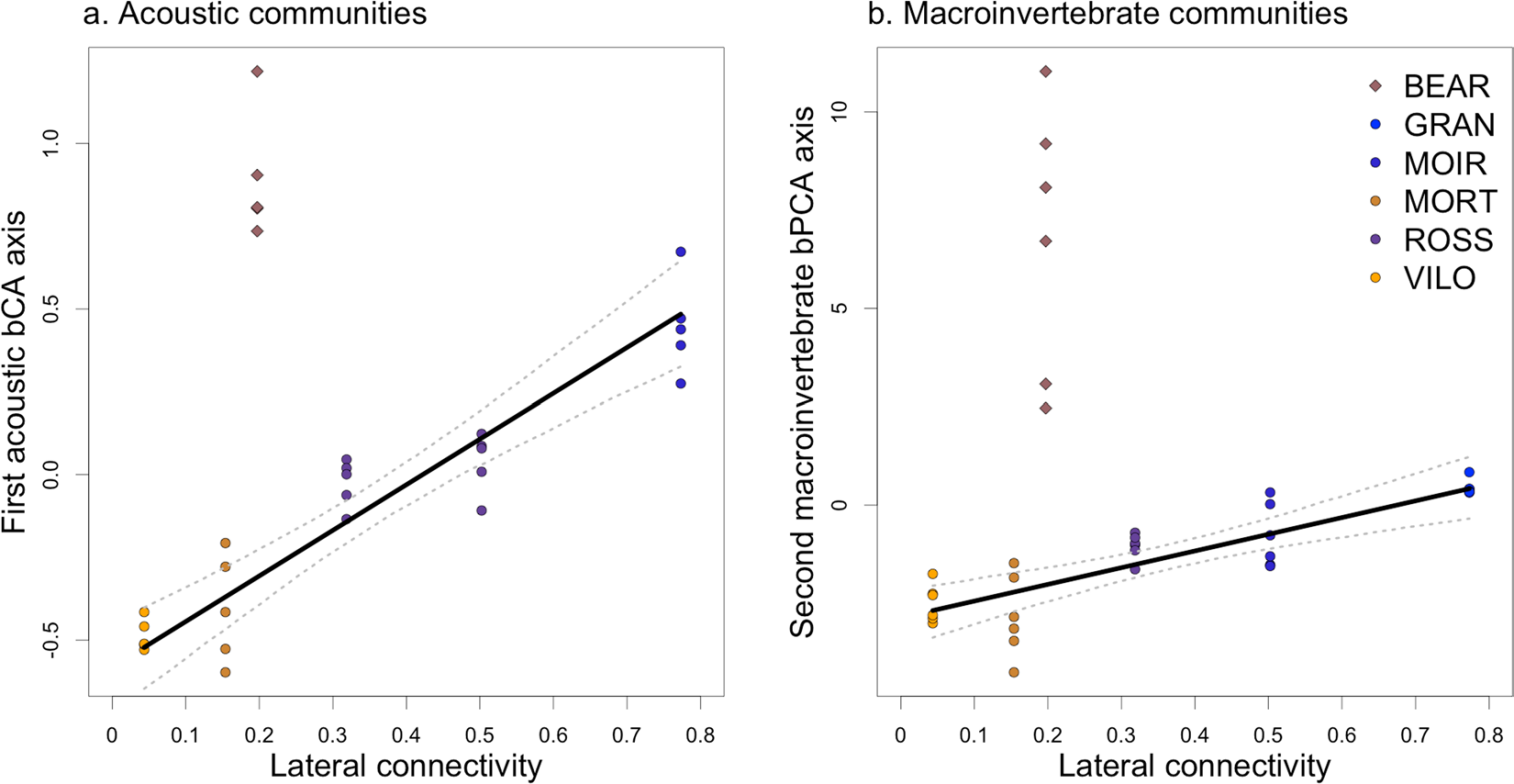
Relationship between the first bCA axis based on acoustic composition and lateral connectivity (**a**); and the second bPCA axis based on macroinvertebrate composition and lateral connectivity (**b**). Each point represents the composition of the acoustic community recorded at one site during one day (**a**) or a sample of macroinvertebrate in one site (**b**). The plain grey line shows the fitted model, excluding the site BEAR. The dotted lines are the 95% confidence interval.

An inspection of the models characteristics revealed a high random intercept for BEAR in model 1 and 3 (Table S4). In addition, the inspection of the bCA space highlighted the outlier position of this site (Fig. 2). Thus, when excluding BEAR, GLMMs identified highly significant relationships between the first bCA axis and lateral connectivity (df = 1, χ^2^ = 14.74, p-value < 0.001, Fig. 4a) and between the third bCA axis and lateral connectivity (df = 1, χ^2^ = 12.33, p-value < 0.001). This model also uncovered a relationship between the third bCA axis and average site temperature (df = 1, χ^2^ = 3.34, p-value < 0.05). None of the environmental variables were associated to the second axis of the bCA (overall model significance: df = 3, χ^2^ = 0.49, p-value = 0.92).

### Link between macroinvertebrate composition and environmental variables

The macroinvertebrate community structure explained by the second and third bPCA axes was not linked to any of the environmental variables, as shown by the GLMMs testing the macroinvertebrate community structure in relation with average site temperature and lateral connectivity (overall model significance for the second bPCA axis: df = 2, χ^2^ = 1.25, p-value = 0.54; and for the third bPCA axis: df = 2, χ^2^ = 2.95, p-value = 0.23; Table 3). In contrast, the first bPCA axis was significantly correlated with average site temperature (df = 1, χ^2^ = 7.00, p-value < 0.01, Table 3).

**Table 3:** Results of the six GLMMs based on macroinvertebrate communities.

Response variable	Term	Estimate	Standard error	Statistics (χ^2^)	Degrees of freedom	P-value
Axis 1	Intercept	0.00	0.65	^(1)^	^(1)^	^(1)^
	Lateral connectivity	1.10	0.86	1.44	1	0.230
	Average temperature	−3.64	0.91	7.00	1	0.008**
Axis 1 excluding BEAR	Intercept	0.69	0.45	^(1)^	^(1)^	^(1)^
	Lateral connectivity	−2.04	0.56	5.62	1	0.02*
	Average temperature	−4.49	0.67	7.18	1	0.007**
Axis 2	Intercept	0.00	1.22	^(1)^	^(1)^	^(1)^
	Lateral connectivity	−0.95	1.60	0.34	1	0.559
	Average temperature	−1.85	1.60	1.21	1	0.272
Axis 2 excluding BEAR	Intercept	−1.46	0.17	^(1)^	^(1)^	^(1)^
	Lateral connectivity	1.05	0.22	8.37	1	0.004**
	Average temperature	−0.05	0.23	0.05	1	0.82
Axis 3	Intercept	0.00	0.92	^(1)^	^(1)^	^(1)^
	Lateral connectivity	1.95	1.14	2.40	1	0.121
	Average temperature	0.44	1.11	0.16	1	0.691
Axis 3 excluding BEAR	Intercept	0.08	1.13	^(1)^	^(1)^	^(1)^
	Lateral connectivity	1.84	1.38	1.52	1	0.218
	Average temperature	0.34	1.34	0.07	1	0.797

For each model and each term in the models, the estimate, the standard error, the χ^2^, the number of degrees of freedom and the p-values are reported, except for intercepts (p-value * < 0.05, ** < 0.01, *** < 0.001). For statistical details, see subsection *Link between acoustic and macroinvertebrate composition and environmental variables* of the Materials and Methods.

^(1)^Not shown due to the lack of meaningful interpretation.

Similarly to acoustic communities, an inspection of the models characteristics revealed a high random intercept for BEAR in model 1 and 2 (Table S5). Thus, when excluding BEAR, GLMMs identified highly significant relationships between the first bPCA axis and lateral connectivity (df = 1, χ^2^ = 5.62, p-value < 0.05); and between the second bPCA axis and the lateral connectivity (df = 1, χ^2^ = 8.37, p-value < 0.01, Fig. 4b). This model also uncovered a relationship between the first bPCA axis and average site temperature (df = 1, χ^2^ = 7.18, p-value < 0.01). None of the environmental variables were associated to the third axis of the bPCA (overall model significance: df = 2, χ^2^ = 2.20, p-value = 0.33).

## Discussion

The diversity and composition of acoustic communities in six secondary channels of the Rhône floodplain could be characterized and distinguished with a rather reasonable sampling effort and without applying taxonomic identification. The underwater acoustic survey conducted over 15 days revealed an important diversity across these communities, composed of 128 different sound types within seven categories. Thus, secondary channels host a remarkable underwater acoustic diversity, as well as other freshwater habitats previously sampled^10^. The most diversified category of sounds recorded was the *composed pulse*, a temporal structure that is one of the most common for acoustic signals produced by aquatic insects^9^. The sound types the most often encountered were *simple pulses* and *irregular sounds*. These categories of sounds are likely to be the by-product of movement or feeding behaviours of several macroinvertebrates taxa. It therefore suggests that a high diversity of the recorded sounds may be emitted by macroinvertebrates.

The 128 sound types inventoried were not randomly distributed among the six monitored floodplain channels, but site-specific as testified by the multi-variate analysis and the low percentage of sound types (15%) shared by all the sites. Thus, each site can be seen as having its own specific acoustic signature over the five days studied. This acoustic diversity pattern is in agreement with the occurrence of different freshwater macroinvertebrate communities in each channel, which showed significant between-channel variations in community composition. The drivers of these singular acoustic signatures can be sought in a series of proximate and ultimate factors hereafter considered successively.

Temperature strongly limits the appropriate conditions for the performance of basic eco-physiological and behavioural functions, including communicating with sound according to species thermal tolerances. The occurrence and activity of soniferous ectotherms, terrestrial or aquatic, may therefore be influenced by environmental temperature, each species occupying a determined thermal niche^28^. Here, the within-site water temperature was rather stable, with variations ranging around 1.5 °C during the study period, implying a restricted or non-existent effect of temperature within each acoustic community over the duration of the study. On the contrary, the substantial thermal differences observed between sites, with variation in average temperatures ranging around 7 °C, contributed to the differences in composition between the acoustic communities. The third bCA axis for acoustic community, and the first bPCA axis for macroinvertebrate community were both correlated with average site temperature. This effect was slightly less important for the acoustic community than for the macroinvertebrate community. Thus, the effect of temperature on the macroinvertebrate composition was also observed by studying the sounds produced by these macroinvertebrates. This suggests that the use of such sounds could be a suitable proxy of macroinvertebrate community composition.

Moreover, a strong linear relationship was found here between composition of acoustic communities and lateral connectivity indicating that the composition of acoustic community progressively changes according to lateral connectivity. Indeed, Castella *et al*. (2015) emphasised lateral connectivity as the major factor shaping the patterns of macroinvertebrate communities. Therefore, the congruence of acoustic and macroinvertebrate community structure with respect to temperature and connectivity supports that the main emitters of sounds in floodplain channel are macroinvertebrates. Furthermore, this finding implies once again that freshwater macroinvertebrate communities and freshwater acoustic communities are comparable in terms of both their composition and their relationship with key ecological factors. In our results, the relative importance of lateral connectivity and temperature is suggested to vary between these two types of communities, with thermal variables playing a more significant role in shaping macroinvertebrate communities. This confirms recent findings based upon a higher number of sites and longer temperature time-series^29^. Given the current state of knowledge, reasons why acoustic communities appear to be more controlled by lateral connectivity remains unknown.

The observed linear relationship between community turnover and lateral connectivity was found to be significant or stronger when removing the site BEAR from the analysis. The outlier position of this site, both in the acoustic and taxonomic analyses, conforms with its location in the floodplain, which makes it more influenced by a hillslope tributary, the Séran, than by the Rhône itself, both in terms of surface and groundwater supply. Riquier *et al*.^22^ also found BEAR to have peculiar sedimentological patterns, the site being “not yet adjusted to new conditions” induced by fluvial restoration. This singularity was also reflected in the macroinvertebrate community that was reported by Paillex *et al*.^21^ as being extremely dense and taxa-rich. BEAR also harbours taxa such as the mayfly *Siphlonurus aestivalis*, only found in BEAR, among 50 floodplain sites monitored along the French Rhône catchment (unpublished data from the Rhône restoration program). We therefore consider the joint identification, both by the taxonomic and acoustic communities, of the BEAR site as departing from the general relationship with lateral connectivity as evidence supporting the congruence between the two types of communities.

Beyond environmental temperature and lateral connectivity, other environmental parameters might have a role in both within and between-community patterns. The acoustic adaptation hypothesis (AAH) suggests that the environment shapes the features of sound signals as a filter retaining only the signals adapted to the environment^30^. According to the AAH, sites having similar propagation properties would lead to sound types showing shared features. This could be the case for the frequency properties of the 128 sound types identified across the floodplain channels. An important fraction of the sound types (77% including the two most abundant categories *simple pulses* and *irregular sounds*) were atonal and half of them covered a specific bandwidth between 2 and 9 kHz with a mean around 5.5 kHz. This shared frequency feature could constitute a variation of Morton’s window defined for forest habitats^30^: sound propagation in these water bodies might be more efficient in the 2–9 kHz frequency range. Such assumption still needs to be verified by conducting appropriate experiments that define the local sound propagation properties. Studying these acoustic properties is a real challenge in these heterogeneous and dynamic environments where sound propagation is far from simple and linear. The depth of the water body is the only factor that has already been considered. Shallow water environments are known to act as a high-pass frequency filter whose cut-off frequency depends on water depth^31^. Here, the average depth of the channels was around 50 cm leading to a cut-off frequency of approximately 2.5 kHz in soft sediment and leaf litter bottom habitats according to the propagation model proposed by Forrest *et al*.^31^. This theoretical value fits well with the lower frequency limit of the 2–9 kHz bandwidth such that the structure of the environment might explain, at least partially, the main frequency feature of the sound recorded.

If the AAH can explain shared acoustic properties, it can also be invoked to explain differences among communities if these communities evolved in distinct environments. Lateral connectivity is an environmental variable discriminating between different riverine habitat types. Obvious differences in ground morphology, sediment nature, and vegetation occur among the studied channels^21,22^. Indeed high lateral connectivity environments have low to no vegetation cover and gravel or rocky bottoms whereas low connectivity environments have generally denser vegetation covers and soft, more organic sediments. These differences could have provided distinct transmission patterns and background noises that may have played a role in the emergence of different communities.

This study identifies for the first time a link between lateral connectivity and acoustic communities. Although the emerging field of ecoacoustics is quickly developing, studies linking habitat variables to acoustics remain scarce^32,33^. This result, in agreement with macroinvertebrate community structure, supports the idea that ecoacoustics can work as a valuable non-invasive alternative to monitor riverine environments and their environmental variables such as connectivity and therefore accessing the complex functioning of the floodplain ecosystem. Acoustic monitoring offers unprecedented opportunities for precise assessment of spatio-temporal dynamics in heterogenous environments such as riverine habitats. The development of real-time monitoring tools is necessary to orient the practitioners’ decisions in such threatened habitats. As already advocated^10^, ecoacoustic investigation opens up new perspectives for the non-invasive and real-time monitoring not only of terrestrial and marine but also of freshwater environments.

## Electronic supplementary material

Supplementary information

## Data Availability

The sounds recorded are archived in the sound library of the Muséum national d’Histoire naturelle and available by inquiry to the corresponding author (cdesjonqu@gmail.com). The raw datasets are archived on github (https://github.com/Desjonqu/srep-18-02685A_data).
