# Supplementary material for: Acoustic communities reflects lateral hydrological connectivity in riverine floodplain similarly to macroinvertebrate communities
Source: Sci Rep. 2018 Sep 26;8:14387. doi: 10.1038/s41598-018-31798-4 (PMC6158236; doi:10.1038/s41598-018-31798-4)

# **Acoustic communities reflects lateral hydrological connectivity in riverine floodplain similarly to macroinvertebrate communities**

Camille Desjonquères[1,2*], Fanny Rybak[2], Emmanuel Castella[3], Diego Llusia[1,2], Jérôme Sueur[1]

[1]Institut de Systématique, Evolution, Biodiversité (ISYEB), Muséum national d'Histoire naturelle, CNRS, Sorbonne Université, EPHE, 57 rue Cuvier, 75005 Paris, France

[2]Neuro-PSI, UMR 9197, Université Paris-Sud, CNRS, Université Paris-Saclay, 91405 Orsay, France

[3]Department F.-A. Forel for Environmental and Aquatic Sciences, Earth and Environmental Science Section and Institute for Environmental Sciences, University of Geneva, 1211 Geneva, Switzerland

* Corresponding author: desjonqu@uwm.edu, Tel.: +33 1 40 79 31 34

Institut de Systématique, Evolution, Biodiversité (ISYEB), Muséum national d'Histoire naturelle, CNRS, Sorbonne Université, EPHE, 57 rue Cuvier, 75005 Paris, France

**Appendix S1: Table S1: Environmental variables in the six sites monitored: geographic location, temperature and connectivity.**

| Site | Geographical position (N/E) | Mean temperature (°C) | Mean absolute daily temperature deviation (°C) | Connectivity |
|---|---|---|---|---|
| BEAR | 45°46'34.118" / 5°46'43.324" | 15.87 | 0.26 | 0.20 |
| GRAN | 45°37'45.647" / 5°38'41.029" | 15.34 | 0.23 | 0.77 |
| MOIR | 45°47'27.342" / 5°46'57.074" | 12.48 | 0.02 | 0.50 |
| MORT | 45°40'1.94" / 5°35'28.952" | 19.71 | 0.70 | 0.15 |
| ROSS | 45°38'13.867" / 5°36'57.227" | 17.90 | 0.5 | 0.32 |
| VILO | 45°38'1.858" / 5°37'10.301" | 18.60 | 0.64 | 0.043 |

**Appendix S2: Table S2: Acoustic characteristics of the 128 recorded sound types: Category, number of sounds analysed, dominant frequency and duration given as mean +/- s.d.** See Table 1 for category description. The four following sound types had a frequency modulation: 28 (-1 kHz), 50 (+1 kHz then -2 kHz), and 83 (-2 kHz).

| Category | Sound type | Number of sounds analysed | Dominant frequency (+/- kHz) | Duration (+/- s) |
|---|---|---|---|---|
| 1. Pure tone | 6 | 1 | 3.871 | 0.021 |
| 1. Pure tone | 15 | 6 | 7.21 +/- 2.302 | 2.852 +/- 3.117 |
| 1. Pure tone | 39 | 6 | 2.566 +/- 0.282 | 0.4 +/- 0 |
| 1. Pure tone | 51 | 6 | 0.502 +/- 0.025 | 0.35 +/- 0.141 |
| 1. Pure tone | 65 | 3 | 15.469 +/- 0.211 | 0.259 +/- 0.036 |
| 1. Pure tone | 67 | 1 | 1.411 | 0.955 |
| 1. Pure tone | 83 | 3 | 5.4 +/- 0.004 | 0.509 +/- 0.16 |
| 1. Pure tone | 95 | 6 | 9.3 +/- 0.871 | 0.854 +/- 0.608 |
| 1. Pure tone | 101 | 6 | 0.973 +/- 0.059 | 0.135 +/- 0.068 |
| 1. Pure tone | 104 | 6 | 8.934 +/- 0.657 | 0.144 +/- 0.025 |
| 1. Pure tone | 121 | 4 | 4.91 +/- 0.223 | 0.336 +/- 0.349 |
| 1. Pure tone | 126 | 6 | 4.563 +/- 0.547 | 0.053 +/- 0.007 |
| 2. Noisy sound | 4 | 5 | 12.068 +/- 6.688 | 0.015 +/- 0.001 |
| 2. Noisy sound | 12 | 6 | 3.19 +/- 0.114 | 1.147 +/- 0.368 |
| 2. Noisy sound | 13 | 1 | 2.408 | 0.085 |
| 2. Noisy sound | 21 | 6 | 0.365 +/- 0.29 | 0.274 +/- 0.1 |
| 2. Noisy sound | 23 | 6 | 1.263 +/- 0.463 | 0.288 +/- 0.184 |
| 2. Noisy sound | 26 | 3 | 2.027 +/- 0.626 | 0.018 +/- 0.003 |
| 2. Noisy sound | 42 | 6 | 1.952 +/- 0.212 | 0.302 +/- 0.082 |
| 2. Noisy sound | 43 | 6 | 1.376 +/- 1.279 | 0.228 +/- 0.066 |
| 2. Noisy sound | 45 | 6 | 0.128 +/- 0.049 | 10 +/- 0 |
| 2. Noisy sound | 46 | 6 | 16.724 +/- 1.24 | 0.1 +/- 0.048 |
| 2. Noisy sound | 52 | 2 | 10.057 +/- 0.153 | 0.052 +/- 0.02 |
| 2. Noisy sound | 77 | 6 | 3.652 +/- 0.594 | 0.048 +/- 0.034 |
| 2. Noisy sound | 89 | 6 | 1.373 +/- 0.096 | 0.8 +/- 0.433 |
| 2. Noisy sound | 90 | 6 | 6.207 +/- 3.121 | 0.252 +/- 0.093 |
| 2. Noisy sound | 91 | 1 | 0.946 | 0.213 |
| 2. Noisy sound | 93 | 6 | 2.037 +/- 0.741 | 0.101 +/- 0.059 |
| 2. Noisy sound | 99 | 3 | 4.293 +/- 0.077 | 0.126 +/- 0.033 |
| 2. Noisy sound | 103 | 6 | 2.316 +/- 0.478 | 0.136 +/- 0.036 |
| 2. Noisy sound | 105 | 6 | 7.111 +/- 1.231 | 0.316 +/- 0.094 |
| 2. Noisy sound | 106 | 6 | 1.151 +/- 0.753 | 0.125 +/- 0.03 |
| 2. Noisy sound | 107 | 2 | 4.021 +/- 0.905 | 0.554 +/- 0.223 |
| 2. Noisy sound | 109 | 6 | 2.487 +/- 0.715 | 0.198 +/- 0.072 |
| 2. Noisy sound | 120 | 1 | 12.972 | 0.134 |
| 2. Noisy sound | 124 | 1 | 7.32 | 0.149 |
| 3. Simple pulse | 7 | 6 | 3.687 +/- 2.211 | 0.016 +/- 0.001 |
| 3. Simple pulse | 11 | 6 | 7.397 +/- 3.395 | 0.018 +/- 0.002 |
| 3. Simple pulse | 18 | 1 | 1.468 | 0.39 |
| 3. Simple pulse | 25 | 6 | 12.639 +/- 3.659 | 0.014 +/- 0.001 |

| | | | | |
|---|---|---|---|---|
| 3. Simple pulse | 44 | 6 | 4.071 +/- 0.492 | 0.018 +/- 0.002 |
| 3. Simple pulse | 64 | 6 | 1.429 +/- 1.191 | 0.016 +/- 0.001 |
| 3. Simple pulse | 75 | 6 | 4.95 +/- 2.881 | 0.018 +/- 0.004 |
| 4. Composed pulse | 1 | 6 | 3.484 +/- 0.325 | 0.116 +/- 0.007 |
| 4. Composed pulse | 10 | 1 | 5.734 | 0.481 |
| 4. Composed pulse | 14 | 4 | 8.212 +/- 2.85 | 1.184 +/- 0.672 |
| 4. Composed pulse | 16 | 6 | 8.049 +/- 1.516 | 3.051 +/- 3.671 |
| 4. Composed pulse | 17 | 6 | 13.387 +/- 2.082 | 0.75 +/- 0.514 |
| 4. Composed pulse | 27 | 6 | 9.484 +/- 0.766 | 1.428 +/- 0.828 |
| 4. Composed pulse | 29 | 6 | 4.51 +/- 1.76 | 0.367 +/- 0.067 |
| 4. Composed pulse | 31 | 6 | 3.413 +/- 0.085 | 0.156 +/- 0.024 |
| 4. Composed pulse | 36 | 1 | 6.245 | 0.91 |
| 4. Composed pulse | 37 | 6 | 9.771 +/- 2.162 | 0.051 +/- 0.019 |
| 4. Composed pulse | 38 | 6 | 11.566 +/- 0.63 | 0.196 +/- 0.021 |
| 4. Composed pulse | 49 | 6 | 8.796 +/- 4.544 | 0.323 +/- 0.092 |
| 4. Composed pulse | 53 | 6 | 8.989 +/- 3.344 | 0.296 +/- 0.078 |
| 4. Composed pulse | 56 | 4 | 15.596 +/- 0.08 | 1.434 +/- 0.443 |
| 4. Composed pulse | 58 | 6 | 10.539 +/- 3.72 | 0.286 +/- 0.179 |
| 4. Composed pulse | 60 | 6 | 5.181 +/- 2.606 | 1.172 +/- 1.071 |
| 4. Composed pulse | 61 | 6 | 10.117 +/- 0.216 | 0.102 +/- 0.078 |
| 4. Composed pulse | 66 | 6 | 4.008 +/- 1.176 | 0.383 +/- 0.036 |
| 4. Composed pulse | 68 | 6 | 10.68 +/- 2.027 | 0.203 +/- 0.051 |
| 4. Composed pulse | 69 | 6 | 3.007 +/- 0.594 | 0.36 +/- 0.065 |
| 4. Composed pulse | 70 | 5 | 3.678 +/- 0.507 | 0.025 +/- 0.001 |
| 4. Composed pulse | 71 | 6 | 8.457 +/- 5.079 | 0.178 +/- 0.067 |
| 4. Composed pulse | 72 | 6 | 10.187 +/- 0.156 | 0.124 +/- 0.075 |
| 4. Composed pulse | 73 | 6 | 3.453 +/- 3.008 | 0.606 +/- 0.426 |
| 4. Composed pulse | 76 | 4 | 3.192 +/- 0.761 | 0.132 +/- 0.015 |
| 4. Composed pulse | 78 | 6 | 2.051 +/- 0.646 | 10 +/- 0 |
| 4. Composed pulse | 81 | 1 | 2.683 | 2.044 |
| 4. Composed pulse | 82 | 6 | 3.728 +/- 0.394 | 0.354 +/- 0.291 |
| 4. Composed pulse | 85 | 6 | 1.663 +/- 0.112 | 0.172 +/- 0.176 |
| 4. Composed pulse | 86 | 1 | 12.651 | 2.431 +/- 0 |
| 4. Composed pulse | 94 | 6 | 7.446 +/- 1.791 | 0.174 +/- 0.084 |
| 4. Composed pulse | 96 | 1 | 2.249 | 0.332 |
| 4. Composed pulse | 97 | 1 | 1.043 | 0.112 |
| 4. Composed pulse | 98 | 6 | 5.186 +/- 1.834 | 0.094 +/- 0.048 |
| 4. Composed pulse | 100 | 6 | 3.967 +/- 0.253 | 0.215 +/- 0.023 |
| 4. Composed pulse | 102 | 2 | 6.237 +/- 2.791 | 0.597 +/- 0.52 |
| 4. Composed pulse | 108 | 6 | 1.633 +/- 0.003 | 0.108 +/- 0.07 |
| 4. Composed pulse | 110 | 4 | 4.064 +/- 0.134 | 0.064 +/- 0.019 |
| 4. Composed pulse | 111 | 6 | 10.702 +/- 1.462 | 0.244 +/- 0.139 |
| 4. Composed pulse | 113 | 2 | 3.492 +/- 0.086 | 4.172 +/- 0.769 |
| 4. Composed pulse | 116 | 3 | 3.199 +/- 0.042 | 0.288 +/- 0.021 |
| 4. Composed pulse | 117 | 1 | 1.922 | 0.486 |
| 4. Composed pulse | 119 | 6 | 6.431 +/- 0.302 | 0.256 +/- 0.064 |
| 4. Composed pulse | 125 | 1 | 5.608 +/- 0 | 2.275 |
| 4. Composed pulse | 127 | 1 | 3.728 +/- 0 | 0.476 |
| 5. Harmonic sound | 9 | 6 | 3.428 +/- 0.234 | 0.275 +/- 0.248 |
| 5. Harmonic sound | 20 | 1 | 13.682 | 0.052 |

| | | | | |
|---|---|---|---|---|
| 5. Harmonic sound | 24 | 6 | 13.232 +/- 0.199 | 0.109 +/- 0.033 |
| 5. Harmonic sound | 28 | 4 | 9.056 +/- 0.02 | 0.612 +/- 0.002 |
| 5. Harmonic sound | 30 | 3 | 9.15 +/- 0.108 | 0.043 +/- 0.007 |
| 5. Harmonic sound | 32 | 2 | 15.178 +/- 0.185 | 0.169 +/- 0.05 |
| 5. Harmonic sound | 33 | 6 | 9.44 +/- 2.456 | 0.117 +/- 0.03 |
| 5. Harmonic sound | 34 | 6 | 9.404 +/- 1.035 | 0.928 +/- 0.554 |
| 5. Harmonic sound | 35 | 6 | 6.562 +/- 1.571 | 0.062 +/- 0.019 |
| 5. Harmonic sound | 40 | 6 | 2.36 +/- 0.13 | 0.115 +/- 0.05 |
| 5. Harmonic sound | 47 | 6 | 2.613 +/- 0.109 | 0.107 +/- 0.023 |
| 5. Harmonic sound | 50 | 6 | 6.09 +/- 2.334 | 0.805 +/- 0.34 |
| 5. Harmonic sound | 55 | 6 | 8.37 +/- 0.444 | 0.11 +/- 0.068 |
| 5. Harmonic sound | 84 | 6 | 7.406 +/- 2.582 | 1.506 +/- 0.334 |
| 5. Harmonic sound | 88 | 6 | 8.77 +/- 1.345 | 0.03 +/- 0.012 |
| 5. Harmonic sound | 114 | 1 | 3.276 | 3.738 |
| 5. Harmonic sound | 115 | 6 | 0.564 +/- 0.002 | 0.451 +/- 0.12 |
| 5. Harmonic sound | 122 | 2 | 15.431 +/- 3.111 | 0.251 +/- 0.089 |
| 6. Irregular sound | 2 | 6 | 0.162 +/- 0.149 | 0.046 +/- 0.033 |
| 6. Irregular sound | 3 | 6 | 0.686 +/- 0.428 | 0.023 +/- 0.005 |
| 6. Irregular sound | 5 | 6 | 1.339 +/- 0.8 | 0.018 +/- 0.001 |
| 6. Irregular sound | 8 | 6 | 0.232 +/- 0.126 | 0.1 +/- 0.028 |
| 6. Irregular sound | 41 | 6 | 3.206 +/- 1.451 | 2.361 +/- 0.972 |
| 6. Irregular sound | 54 | 6 | 3.219 +/- 2.987 | 0.022 +/- 0.006 |
| 6. Irregular sound | 57 | 6 | 0.2 +/- 0 | 10 +/- 0 |
| 6. Irregular sound | 62 | 6 | 0.654 +/- 0.44 | 10 +/- 0 |
| 6. Irregular sound | 63 | 6 | 7.652 +/- 4.687 | 0.037 +/- 0.03 |
| 6. Irregular sound | 74 | 6 | 0.497 +/- 0.159 | 10 +/- 0 |
| 6. Irregular sound | 79 | 5 | 0.2 +/- 0.037 | 4.025 +/- 3.235 |
| 6. Irregular sound | 92 | 6 | 5.666 +/- 4.934 | 0.832 +/- 0.042 |
| 7. Composed sound | 19 | 6 | 2.081 +/- 1.007 | 2.545 +/- 1.42 |
| 7. Composed sound | 22 | 6 | 3.731 +/- 2.142 | 0.162 +/- 0.116 |
| 7. Composed sound | 48 | 4 | 11.158 +/- 0.125 | 6.896 +/- 4.602 |
| 7. Composed sound | 59 | 6 | 2.096 +/- 0.635 | 0.609 +/- 0.225 |
| 7. Composed sound | 80 | 6 | 9.933 +/- 1.784 | 3.231 +/- 1.225 |
| 7. Composed sound | 87 | 6 | 6.449 +/- 0.117 | 1.394 +/- 0.135 |
| 7. Composed sound | 112 | 6 | 5.083 +/- 0.03 | 5.276 +/- 0.363 |
| 7. Composed sound | 118 | 6 | 5.644 +/- 0.144 | 4.204 +/- 2.22 |
| 7. Composed sound | 123 | 1 | 13.323 | 0.212 |
| 7. Composed sound | 128 | 1 | 2.405 | 10 |

**Table S3 : Macroinvertebrates found in the six sites.**

| Class | Species or genus or family | BEAR | GRAN | MOIR | MORT | ROSS | VILO |
|---|---|---|---|---|---|---|---|
| Arachnida | Hydrachnidia ind | 1 | 1 | 1 | 1 | 1 | 1 |
| Bivalvia | *Anodonta anatina* | 0 | 1 | 0 | 0 | 0 | 0 |
| Bivalvia | *Corbicula fluminea* | 0 | 1 | 0 | 0 | 0 | 0 |
| Bivalvia | *Musculium lacustre* | 1 | 1 | 0 | 0 | 1 | 1 |
| Bivalvia | Pisidium sp. | 1 | 1 | 1 | 1 | 1 | 1 |
| Bivalvia | Sphaerium sp. | 1 | 1 | 0 | 1 | 1 | 1 |
| Clitelata | Alboglossiphonia sp. | 1 | 0 | 1 | 1 | 1 | 1 |
| Clitelata | *Erpobdella octoculata* | 1 | 0 | 0 | 0 | 1 | 0 |
| Clitelata | Erpobdella sp. | 1 | 0 | 0 | 0 | 1 | 0 |
| Clitelata | Erpobdellidae ind | 1 | 0 | 0 | 0 | 1 | 0 |
| Clitelata | Glossiphonia sp. | 1 | 1 | 1 | 1 | 1 | 1 |
| Clitelata | *Helobdella stagnalis* | 1 | 1 | 1 | 1 | 1 | 1 |
| Clitelata | *Hemiclepsis marginata* | 1 | 1 | 0 | 1 | 1 | 1 |
| Clitelata | *Theromyzon tessulatum* | 0 | 0 | 0 | 0 | 1 | 1 |
| Gastropoda | *Acroloxus lacustris* | 0 | 0 | 0 | 0 | 1 | 1 |
| Gastropoda | *Anisus vortex* | 1 | 1 | 0 | 1 | 1 | 0 |
| Gastropoda | *Anisus vorticulus* | 0 | 0 | 0 | 0 | 1 | 1 |
| Gastropoda | *Bithynia tentaculata* | 1 | 1 | 0 | 1 | 1 | 1 |
| Gastropoda | *Ferrissia clessiniana* | 0 | 0 | 0 | 1 | 1 | 0 |
| Gastropoda | *Gyraulus albus* | 1 | 1 | 0 | 1 | 1 | 0 |
| Gastropoda | *Gyraulus parvus* | 1 | 0 | 1 | 0 | 0 | 1 |
| Gastropoda | *Haitia acuta* | 1 | 1 | 1 | 1 | 1 | 1 |
| Gastropoda | *Hippeutis complanatus* | 1 | 0 | 0 | 1 | 1 | 1 |
| Gastropoda | *Lymnaea stagnalis* | 0 | 0 | 0 | 1 | 1 | 1 |
| Gastropoda | *Physa fontinalis* | 1 | 0 | 0 | 0 | 1 | 0 |
| Gastropoda | Planorbidae ind | 1 | 0 | 0 | 0 | 1 | 1 |
| Gastropoda | *Planorbis carinatus* | 1 | 1 | 1 | 1 | 1 | 1 |
| Gastropoda | *Potamopyrgus antipodarum* | 1 | 1 | 1 | 0 | 0 | 0 |
| Gastropoda | *Radix auricularia* | 1 | 1 | 1 | 0 | 0 | 0 |
| Gastropoda | *Radix balthica* | 1 | 0 | 1 | 0 | 0 | 0 |
| Gastropoda | Radix sp. | 1 | 1 | 1 | 1 | 0 | 1 |
| Gastropoda | Stagnicola sp. | 1 | 0 | 0 | 0 | 0 | 1 |
| Gastropoda | *Valvata cristata* | 1 | 1 | 0 | 1 | 1 | 1 |
| Gastropoda | *Valvata piscinalis* | 1 | 0 | 1 | 0 | 0 | 1 |
| Insecta | Aeshna sp. | 0 | 0 | 1 | 0 | 0 | 0 |
| Insecta | Aeshnidae ind | 0 | 0 | 0 | 1 | 0 | 1 |
| Insecta | *Agabus bipustulatus* | 0 | 0 | 1 | 0 | 0 | 0 |
| Insecta | Agabus sp. | 1 | 0 | 1 | 1 | 0 | 0 |
| Insecta | *Agabus sturmii* | 1 | 0 | 1 | 0 | 0 | 0 |
| Insecta | *Agabus undulatus* | 1 | 0 | 0 | 0 | 0 | 0 |
| Insecta | *Agrypnia varia* | 0 | 0 | 0 | 0 | 0 | 1 |
| Insecta | *Anabolia nervosa* | 0 | 1 | 0 | 0 | 1 | 1 |
| Insecta | Anophelinae ind | 0 | 1 | 1 | 0 | 1 | 1 |
| Insecta | *Athripsodes aterrimus* | 1 | 1 | 1 | 1 | 1 | 1 |
| Insecta | Athripsodes sp. | 1 | 1 | 0 | 0 | 1 | 1 |
| Insecta | *Boyeria irene* | 0 | 0 | 0 | 0 | 0 | 1 |

| | | | | | | | |
|---|---|---|---|---|---|---|---|
| Insecta | *Caenis horaria* | 1 | 1 | 0 | 1 | 0 | 1 |
| Insecta | *Caenis luctuosa* | 0 | 1 | 0 | 0 | 0 | 0 |
| Insecta | *Caenis macrura* | 0 | 1 | 1 | 0 | 0 | 1 |
| Insecta | *Caenis robusta* | 0 | 0 | 0 | 1 | 0 | 1 |
| Insecta | Caenis sp. | 1 | 0 | 0 | 0 | 0 | 1 |
| Insecta | *Cataclysta lemnata* | 0 | 1 | 0 | 0 | 1 | 0 |
| Insecta | Ceratopogonidae ind | 1 | 1 | 1 | 1 | 1 | 1 |
| Insecta | Chaoborus sp. | 0 | 1 | 0 | 1 | 1 | 1 |
| Insecta | Chironomidae ind | 1 | 1 | 1 | 1 | 1 | 1 |
| Insecta | Chrysomelidae ind | 1 | 0 | 0 | 0 | 0 | 0 |
| Insecta | *Cloeon dipterum* | 1 | 1 | 1 | 1 | 1 | 1 |
| Insecta | Coenagrion sp. | 1 | 1 | 1 | 1 | 1 | 1 |
| Insecta | Coenagrionidae ind | 1 | 1 | 1 | 1 | 1 | 1 |
| Insecta | Corixinae ind | 1 | 1 | 1 | 1 | 1 | 1 |
| Insecta | Culicidae ind | 1 | 0 | 1 | 0 | 1 | 0 |
| Insecta | *Dasyheleinae* | 1 | 1 | 0 | 1 | 1 | 1 |
| Insecta | Dolichopodidae ind | 1 | 0 | 0 | 0 | 0 | 0 |
| Insecta | Dytiscidae ind | 0 | 0 | 1 | 0 | 0 | 0 |
| Insecta | Dytiscus sp. | 0 | 0 | 1 | 0 | 0 | 0 |
| Insecta | Elmis sp. | 1 | 0 | 0 | 0 | 0 | 0 |
| Insecta | Elophila sp. | 0 | 0 | 0 | 1 | 0 | 0 |
| Insecta | Ephydridae ind | 1 | 0 | 1 | 0 | 0 | 0 |
| Insecta | *Erythromma viridulum* | 0 | 0 | 0 | 0 | 0 | 1 |
| Insecta | Esolus sp. | 0 | 0 | 1 | 0 | 0 | 0 |
| Insecta | Gerris sp. | 0 | 1 | 0 | 0 | 0 | 0 |
| Insecta | *Glyphotaelius pellucidus* | 0 | 0 | 1 | 0 | 0 | 1 |
| Insecta | Graptodytes sp. | 1 | 0 | 0 | 0 | 0 | 0 |
| Insecta | Haliplidae ind | 0 | 0 | 0 | 0 | 1 | 0 |
| Insecta | *Haliplus flavicollis* | 1 | 0 | 1 | 0 | 0 | 0 |
| Insecta | *Haliplus fluviatilis* | 1 | 1 | 1 | 1 | 1 | 0 |
| Insecta | *Haliplus laminatus* | 1 | 1 | 0 | 1 | 0 | 0 |
| Insecta | *Haliplus lineatocollis* | 0 | 0 | 1 | 0 | 0 | 1 |
| Insecta | *Haliplus obliquus* | 0 | 0 | 0 | 1 | 1 | 0 |
| Insecta | *Haliplus ruficollis* | 0 | 0 | 0 | 1 | 0 | 1 |
| Insecta | Haliplus sp. | 0 | 1 | 1 | 1 | 1 | 0 |
| Insecta | Haliplus sp. | 1 | 1 | 1 | 1 | 1 | 1 |
| Insecta | Helochares sp. | 1 | 0 | 0 | 0 | 0 | 0 |
| Insecta | *Helophorus minutus* | 0 | 0 | 1 | 0 | 0 | 0 |
| Insecta | Hemerodromia sp. | 0 | 0 | 1 | 0 | 0 | 0 |
| Insecta | *Hydaticus seminiger* | 1 | 0 | 0 | 0 | 0 | 0 |
| Insecta | *Hydraena melas* | 1 | 0 | 1 | 0 | 0 | 0 |
| Insecta | *Hydraena palustris* | 0 | 0 | 1 | 0 | 0 | 0 |
| Insecta | Hydraena sp. | 1 | 0 | 1 | 0 | 0 | 0 |
| Insecta | *Hydrochara caraboides* | 1 | 0 | 0 | 0 | 0 | 0 |
| Insecta | *Hydroporus palustris* | 0 | 0 | 1 | 1 | 0 | 0 |
| Insecta | Hydroporus sp. | 1 | 1 | 1 | 1 | 0 | 0 |
| Insecta | Hydroptila sp. | 0 | 0 | 1 | 0 | 0 | 0 |
| Insecta | *Hygrotus impressopunctatus* | 0 | 0 | 1 | 0 | 0 | 0 |

| | | | | | | | |
|---|---|---|---|---|---|---|---|
| Insecta | *Hyphydrus ovatus* | 1 | 0 | 0 | 1 | 0 | 0 |
| Insecta | Hyphydrus sp. | 0 | 0 | 0 | 1 | 1 | 0 |
| Insecta | *Ilybius fuliginosus* | 1 | 0 | 0 | 0 | 0 | 0 |
| Insecta | Ilybius sp. | 1 | 0 | 0 | 0 | 0 | 0 |
| Insecta | *Ilyocoris cimicoides* | 1 | 0 | 0 | 1 | 0 | 1 |
| Insecta | *Ischnura elegans* | 0 | 0 | 0 | 1 | 0 | 1 |
| Insecta | Laccobius sp. | 1 | 1 | 0 | 0 | 0 | 0 |
| Insecta | Laccophilus sp. | 1 | 1 | 1 | 0 | 1 | 0 |
| Insecta | *Leptocerus tineiformis* | 0 | 0 | 0 | 0 | 0 | 1 |
| Insecta | *Lestes viridis* | 0 | 0 | 1 | 0 | 1 | 0 |
| Insecta | Leuctra sp. | 0 | 0 | 1 | 0 | 0 | 0 |
| Insecta | *Limnephilus flavicornis* | 0 | 0 | 0 | 1 | 0 | 1 |
| Insecta | *Limnephilus germanus* | 1 | 0 | 1 | 0 | 0 | 1 |
| Insecta | *Limnephilus lunatus* | 1 | 1 | 1 | 1 | 1 | 0 |
| Insecta | *Limnephilus nigriceps* | 0 | 0 | 0 | 1 | 0 | 0 |
| Insecta | Limnephilus sp. | 1 | 0 | 0 | 0 | 1 | 1 |
| Insecta | Micronecta sp. | 0 | 1 | 0 | 0 | 0 | 0 |
| Insecta | Microvellia sp. | 1 | 0 | 0 | 0 | 0 | 0 |
| Insecta | Muscidae ind | 0 | 0 | 0 | 1 | 0 | 0 |
| Insecta | *Mystacides azurea* | 1 | 1 | 1 | 0 | 0 | 0 |
| Insecta | *Mystacides longicornis* | 1 | 0 | 0 | 0 | 0 | 0 |
| Insecta | Mystacides sp. | 0 | 0 | 0 | 0 | 0 | 1 |
| Insecta | Notonecta sp. | 0 | 0 | 1 | 0 | 0 | 0 |
| Insecta | Oulimnius sp. | 0 | 1 | 0 | 0 | 0 | 0 |
| Insecta | *Parapoynx stratiotata* | 0 | 0 | 0 | 1 | 0 | 0 |
| Insecta | *Peltodytes caesus* | 1 | 0 | 0 | 0 | 0 | 0 |
| Insecta | Peltodytes sp. | 0 | 1 | 0 | 0 | 0 | 0 |
| Insecta | Pilaria sp. | 1 | 0 | 0 | 0 | 0 | 0 |
| Insecta | Platycnemis cf. pennipes | 1 | 1 | 0 | 0 | 0 | 0 |
| Insecta | *Plea minutissima* | 1 | 0 | 0 | 1 | 1 | 1 |
| Insecta | *Potamanthus luteus* | 1 | 1 | 1 | 0 | 1 | 1 |
| Insecta | *Pyrrhosoma nymphula* | 1 | 0 | 1 | 0 | 0 | 0 |
| Insecta | *Sialis lutaria* | 1 | 1 | 1 | 1 | 1 | 1 |
| Insecta | *Siphlonorus aestivalis* | 1 | 0 | 0 | 0 | 0 | 0 |
| Insecta | *Stictotarsus duodecimpustulatus* | 0 | 1 | 1 | 0 | 0 | 0 |
| Insecta | Stictotarsus sp. | 0 | 1 | 1 | 0 | 0 | 0 |
| Insecta | Stratiomyidae ind | 1 | 1 | 1 | 1 | 1 | 1 |
| Insecta | Sympetrum sp. | 1 | 1 | 0 | 0 | 0 | 0 |
| Insecta | Tabanidae ind | 1 | 1 | 0 | 1 | 1 | 0 |
| Insecta | Tipulidae ind | 1 | 1 | 0 | 0 | 0 | 0 |
| Malacostraca | *Asellus aquaticus* | 1 | 1 | 1 | 1 | 1 | 1 |
| Malacostraca | *Crangonyx pseudogracilis* | 1 | 0 | 1 | 1 | 0 | 0 |
| Malacostraca | *Gammarus pulex/fossarum* | 1 | 1 | 1 | 1 | 0 | 0 |
| Turbellaria | Dugesia/Planaria sp. | 1 | 1 | 1 | 1 | 1 | 1 |
| Turbellaria | *Girardia tigrina* | 1 | 0 | 1 | 0 | 0 | 0 |
| Turbellaria | *Polycelis nigra/tenuis* | 1 | 1 | 1 | 0 | 0 | 0 |
| Turbellaria | Polycelis sp. | 1 | 1 | 1 | 0 | 0 | 0 |
| Turbellaria | Turbellaria ind | 1 | 0 | 1 | 1 | 0 | 1 |

**Appendix S4: Table S4: Random intercept values for the sites in the models 1 and 3 based on acoustic composition.** The models were Generalized Linear Mixed Models (GLMM) designed to analyse the relationship between the acoustic composition of the sites and the environmental variables.

| Site | Random intercept model 1 | Random intercept model 2 | Random intercept model 3 |
|---|---|---|---|
| BEAR | **0.77** | -0.11 | **0.31** |
| GRAN | 0.14 | -0.37 | 0.005 |
| MOIR | -0.45 | 0.25 | -0.07 |
| MORT | -0.16 | 0.40 | -0.03 |
| ROSS | 0.01 | 0.42 | -0.01 |
| VILO | -0.31 | -0.59 | -0.25 |

Appendix S5: Table S5: **Random intercept values for the sites in the models 1 and 2 based on macroinvertebrate composition**. The models were Generalized Linear Mixed Models (GLMM) designed to analyse the relationship between the macroinvertebrate composition of the sites and the environmental variables.

| **Site** | **Random intercept model 1** | **Random intercept model 2** | **Random intercept model 3** |
|---|---|---|---|
| BEAR | **-2.42** | **5.41** | -0.29 |
| GRAN | -0.95 | 1.09 | 1.06 |
| MOIR | 1.77 | -3.18 | -1.02 |
| MORT | 1.28 | -1.14 | 3.05 |
| ROSS | 0.40 | -0.15 | 0.29 |
| VILO | -0.08 | -2.03 | 3.03 |

Appendix S6: Figure S1: **Map showing the location of the study sites.** General localization in France mainland and local position of the sites using an OpenStreetMap map tile (© OpenStreetMap, www.opendatacommons.org/copyright). The cartography in the OpenStreetMap map tiles is licensed under CC BY-SA (www.openstreetmap.org/copyright). The licence terms can be found on the following link: [http://creativecommons.org/licenses/by-sa/2.0/](http://creativecommons.org/licenses/by-sa/2.0/).

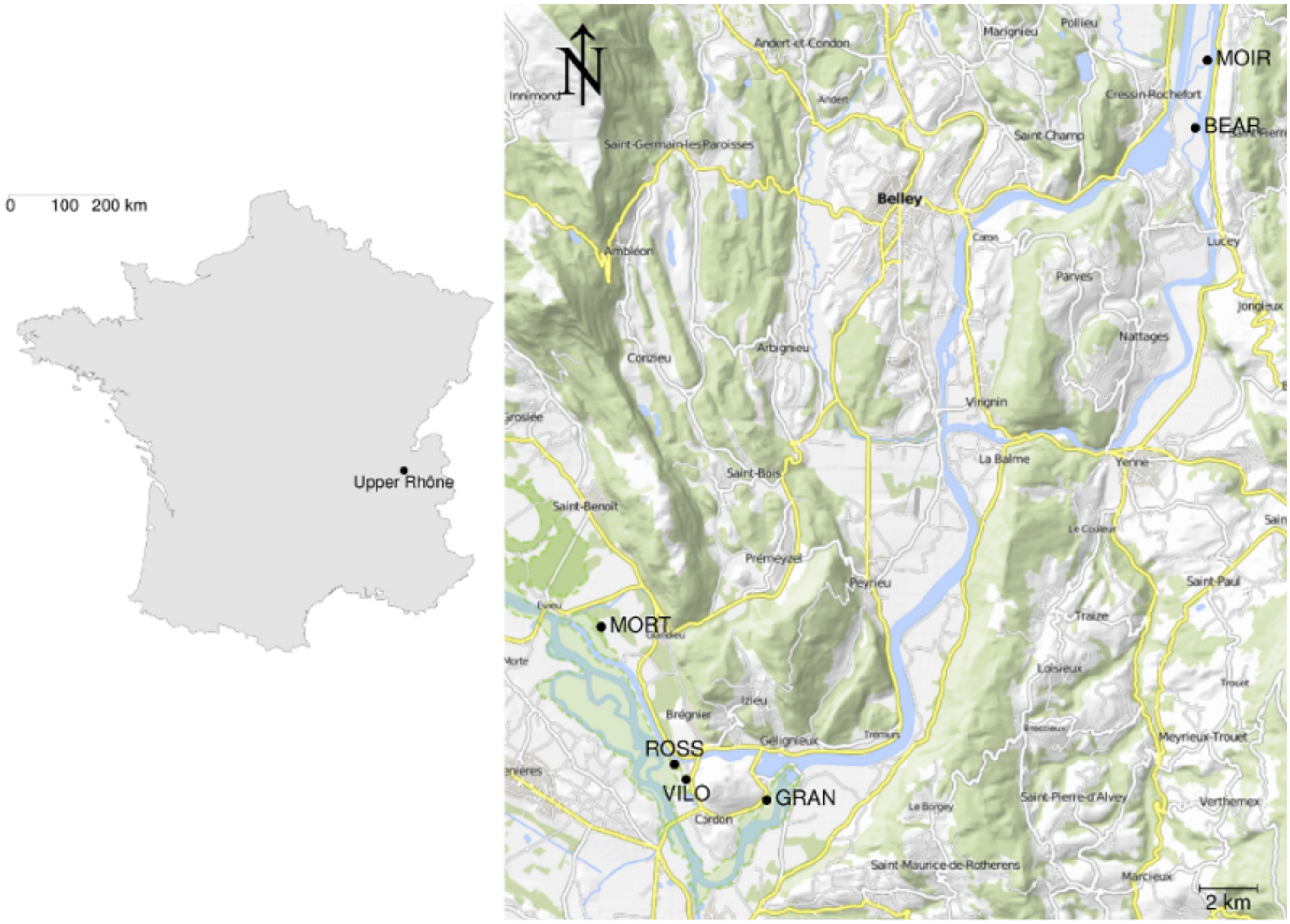

Appendix S7: Figure S2: **Main properties of the seven sound type categories.** The dispersion of sound dominant frequency (Hz) and duration (s) are shown with 95% confidence interval segments. The intersection of the segments corresponds to the mean. Sample sizes: 54, 109, 37, 205, 85, 71, and 48 for the categories from 1 to 7, respectively. See Table 1 for category description.

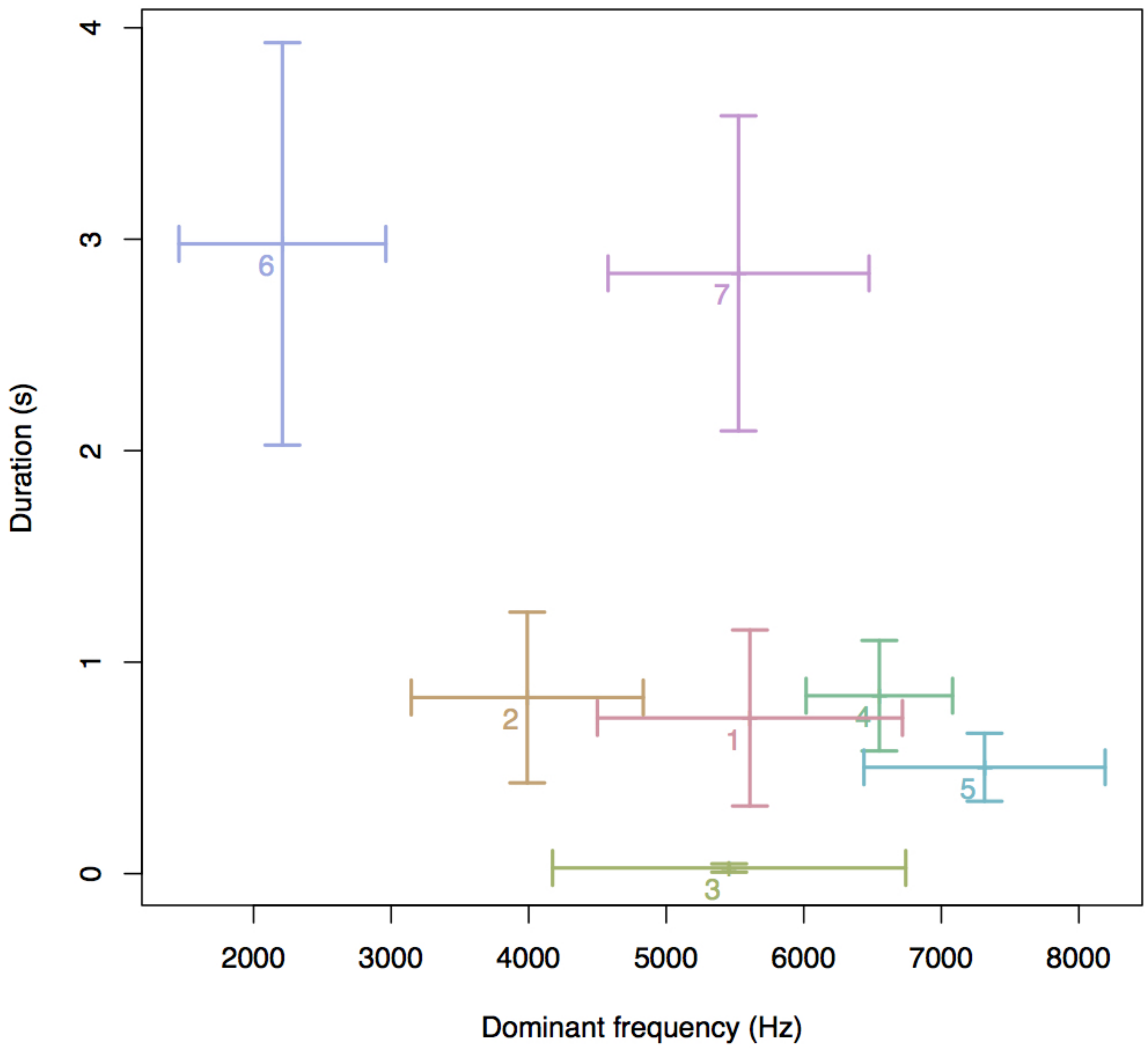

Supplement: Supplementary file 1 — Supplementary information [file 41598_2018_31798_MOESM1_ESM.pdf]
